# Identification and Characterization of lncRNAs Expression Profile Related to Goat Skeletal Muscle at Different Development Stages

**DOI:** 10.3390/ani12192683

**Published:** 2022-10-06

**Authors:** Haiyin Han, Xianwei Wang, Wentao Li, Jiannan Liu, Yekai Fan, Hui Zhang, Junqi Yang, Yahui Gao, Yufang Liu

**Affiliations:** 1 School of Life Sciences and Food Engineering, Hebei University of Engineering, Handan 056021, China; 2 Henan Animal Husbandry Service, Zhengzhou 450046, China; 3 School of Landscape and Ecological Engineering, Hebei University of Engineering, Handan 056021, China

**Keywords:** goat, skeletal muscle, lncRNAs, RNA sequencing, regulation networks

## Abstract

**Simple Summary:**

The skeletal muscle growth and development affects the production of goat meat. Here, we performed transcriptome sequencing of longissimus dorsi muscle of goats at two development stages (namely, 1-month-old and 9-month-old) by RNA sequencing, assessing the lncRNA expression profile during skeletal muscle development. This study identified regulatory lncRNAs and pathways related to the development of skeletal muscle, providing a reference for future study on the molecular mechanism that regulates the skeletal growth and development.

**Abstract:**

LncRNAs are essential for regulating skeletal muscle. However, the expression profile and function of lncRNAs in goat muscle remains unclear. Here, an average of ~14.58 Gb high-quality reads were obtained from longissimus dorsi tissues of 1-month-old (n = 3) and 9-month-old (n = 3) Wu’an black goats using RNA sequencing. Of a total of 3441 lncRNAs, 1281 were lincRNAs, 805 were antisense lncRNAs, and 1355 were sense_overlapping lncRNAs. These lncRNAs shared some properties with goats, such as fewer exons, shorter transcript, and open reading frames (ORFs) length. Among them, 36 differentially expressed lncRNAs (DE lncRNA) were identified, and then 10 random lncRNAs were validated by RT-qPCR. Furthermore, 30 DE lncRNAs were neighboring 71 mRNAs and several genes were functionally enriched in muscle development-related pathways, such as *APC*, *IFRD1*, *NKX2-5*, and others. Additionally, 36 DE lncRNAs and 2684 mRNAs were included in co-expression interactions. A lncRNA-miRNA-mRNA network containing 4 lncRNAs, 3 miRNAs, and 8 mRNAs was finally constructed, of which XR_001296113.2 might regulate *PDLIM7* expression by interaction with chi-miR-1296 to affect skeletal muscle development. This study revealed the expression profile of goat lncRNAs for further investigative studies and provides a fuller understanding of skeletal muscle development.

## 1. Introduction

As an important economically farm animal, the goat is raised for the utilization of meat, cashmere, and milk. With people’s living standards having improved, the demand for goat meat has gradually increased. As a result, the lower meat production has hindered the development of the goat industry. Postnatal muscle growth is positively correlated with muscle fiber diameter, with larger muscle fiber diameters resulting in faster muscle growth rates [1,2]. Recently, the studies of genome-wide transcription have provided a series of valuable candidate genes that regulate muscle growth in goats [3,4]. Thus, uncovering the genetic mechanisms underneath muscle growth could help us to improve the meat production. The Wu’an black goat, a unique black goat breed in the Hebei province of China, has attracted increasing interest due to its better meat performance. This goat breed has many outstanding characteristics such as good meat quality, rich protein content, low fat and cholesterol content, and good flavor, which has high breeding value [5,6]. LncRNAs are transcribed as RNAs longer than 200 bp in length, with a complex structure and no protein-coding ability [7], but can regulate gene expression and are involved in multiple physiological processes, including cell proliferation, differentiation, and apoptosis [8]. They are widely distributed in different species, such as animals, plants, yeast, and even viruses [9,10,11,12]. Moreover, lncRNAs are expressed temporally, spatially, and tissue-specific expression, and play a vital role in biological processes like transcriptional regulation [13], epigenetic modification [14], and in development [15]. The complicated process of muscle development necessitates the interaction of numerous factors. However, the function of lncRNAs in revealing skeletal muscle growth and development remains unclear, and understanding the molecular mechanism is crucial.

Plenty of lncRNAs have been showed to play a key role in the skeletal muscle development in multiple species, including goats, sheep, and pigs [16,17,18]. LncRNAs have been shown in sheep to have an important regulatory function in muscle growth and development. During muscles growth in Hu sheep, the genome-wide analysis was used to detect the DE lncRNAs of the skeletal muscles at three key development stages (fetus, lamb, and adult). The results showed that 6924 lncRNAs were generated, of which the DE lncRNAs were involved in the essential bio-function and processes, including skeletal muscle development [19]. Furthermore, in-depth analysis of the sequencing data identified the temporal expression patterns of lncRNAs in the sheep longissimus dorsi muscle from gestation to postnatal stages, and described the functional lncRNAs that regulated the development differentiation of the muscle [20]. Similarly, the expression profiles of lncRNAs were verified in ovine (Texel and Ujumqin) gastrocnemius muscle at fetal (days 85 and 120 of gestation), newborn, and adult stage [21]. In recent years, there has been increasing evidence of the important functions of lncRNAs in the skeletal muscle development of goats [10,22]. Transcriptional sequencing analysis of Jianzhou big-eared goats at fetal (45, 60, and 105 days of gestation) and postnatal (3 days after birth) stage showed that 577 DE lncRNAs may play a vital role in skeletal muscle development [23]. The genome-wide studies of goat lncRNAs associated with skeletal muscle development have been carried out. The information about functional lncRNAs in skeletal muscle development is still limited. We hope to elucidate the functional mechanisms of DE lncRNAs in goat skeletal muscle tissue from a new perspective.

Here, we provided the expression pattern and the potential role of the DE lncRNAs from the longissimus dorsi muscle of Wu’an black goat at two developmental stages (kid: 1 month; youth: 9 months) by using RNA sequencing. The biological functions of DE lncRNAs molecules were annotated through GO analysis and KEGG pathway enrichment analysis. Finally, the lncRNA-miRNA-mRNA ceRNA network was constructed to clarify the molecular mechanism underpinning skeletal muscle development. The aim of this study will facilitate a better understanding of transcriptomic changes during skeletal muscle development of goats and provide a reference dataset for future studies on the molecular mechanisms that regulate the skeletal growth and development.

## 2. Materials and Methods

### 2.1. Animal Preparation and Sample Collection 

The animals used in this study were the Wu’an black goats from the Yutian Black Goat farm (Wu’an, China). A total of six health female Wu’an black goats were selected, covering two groups at different growth stages: 1-month-old and 9-month-old. The animal samples (longissimus dorsi) were collected and immediately frozen in liquid nitrogen, and then stored at −80 °C for RNA sequencing analysis.

### 2.2. RNA Isolation, LncRNA-Seq Library Construction, and Sequencing

Total RNA was isolated from the 6 muscle tissue samples using TRIzol reagent (Invitrogen, Carlsbad, CA, USA). Degradation and contamination of the RNA samples were monitored by 1% agarose gel electrophoresis. The purity and concentration of the RNA samples were evaluated by the NanoDrop 2000 spectrophotometer (Thermo Scientific, Wilmington, DE, USA). The integrity of the total RNA was assessed by the Agilent Bioanalyzer 2100 system (Agilent Technologies, Palo Alto, CA, USA) with the RNA Nano 6000 assay kit. The RNA samples with a RNA integrity number (RIN) score greater than 8 were used for sequencing. 

DNA was removed with deoxyribonuclease I (Takara, Dalian, China). Ribosomal RNAs were removed from the total RNA using the Ribo-zero^TM^ Gole Kit (Illumina, San Diego, CA, USA) according to the manufacturer’s instructions. Then, high quality strand-specificity libraries were generated using the rRNA-depleted RNA. Briefly, the rRNA-depleted RNA was broken into short fragments of 250−300 bp. The first strand of cDNA was synthesized using fragmented RNA as a template and random oligonucleotides primers. This was followed by the second strand cDNA synthesis using dNTPs (dUTP, dATP, dGTP, and dCTP), DNA polymerase Ⅰ, and RNase H. The purified double-stranded cDNA then underwent end repair, A-tailing, and ligation of a sequencing adapter. Finally, the products were purified by the AMPure XP system, and an Agilent Bioanalyzer 2100 system was used to assess the library quality. The Illumina Novaseq platform at Novogene (Beijing, China) was used to sequence libraries, and 200 bp paired-end reads were generated.

Raw data in fastq format were processed by in-house Perl script that filtered out reads containing adapter, poly-N, and low-quality reads [24]. Meanwhile, the Phred scores of Q20 and Q30 were calculated, as well as the GC content of the high-quality clean data. After the filtering process, high quality clean reads were obtained. All the downstream analyses were based on the high-quality clean data. The goat reference genome and gene model annotation files were downloaded from the genome website. Then, the high-quality clean reads were aligned to goat reference genome (CHIR_1.0, NCBI) using HISAT2. Based on this, the mapped reads were assembled using StringTie following a reference-based approach. Finally, both known genes and new transcripts from the results of HISAT2 alignment were merged by Cuffmerge program and annotated by Cuffcompare.

### 2.3. Prediction of Multi-Exon LncRNAs

**Figure 1:**
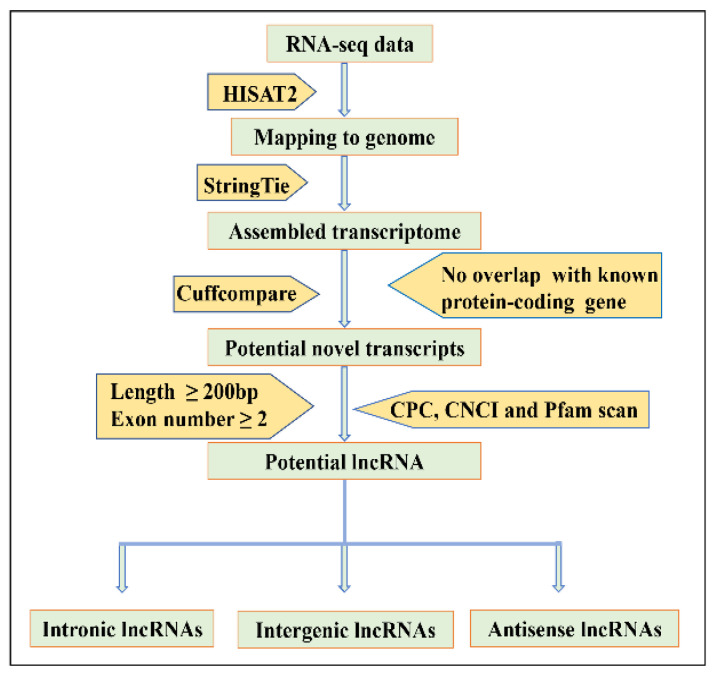
Identification of the putative lncRNAs. The detailed step of filtering pipeline is depicted in the methods section.

The assembled new transcripts from the lncRNA-seq libraries were filtered to acquire the putative lncRNAs. The identification criteria for the putative lncRNA candidates were in line with the pipeline shown in Figure 1. The assembled transcripts were filtered out according to the steps as follows: (1) Low-confidence single exon transcripts or transcripts shorter than 200 bp in length were discarded. (2) To obtain unannotated transcripts, the known transcripts were filtered out using Cuffcompare. (3) The transcripts that contained protein-coding potential were also filtered. The software programs of Coding Potential Calculator [25] and Coding-Non-Coding Index [26], as well as Protein Families Database [27], were used to predict the coding potential of transcripts. Only transcripts without predicted protein-coding potential remained as putative lncRNAs.

### 2.4. Classification and Differential Expression Analysis

The potential lncRNAs were classified based on their association with the annotated protein-coding genes. In this study, the association between the potential lncRNAs and the annotated protein-coding genes was compared with cuffmerge [28]. Then, the classification and characteristics of lncRNAs were analyzed. StringTie was selected to analyze the expression levels of lncRNAs by calculating fragments per kilobase million (FPKM). The corrected *p*-value < 0.05 and an absolute value of the |log2FoldChange| ≥ 2 were set as the threshold to assess statistically significant differences of lncRNA expression.

### 2.5. Co-Location and Co-Expression Analysis, and Functional Annotation Analysis

LncRNAs exert cis-regulatory effects on their co-localized genes [13]. However, lncRNAs regulate the expression of genes located on other chromosomes through a trans-acting mechanism [29]. To assess the potential function of DE lncRNAs, we predicted the possible target genes that were co-located and co-expressed with lncRNAs. In this study, coding genes located 100 kb/100 kb of DE lncRNA were classified as putative cis-acting targets. The trans-acting correlations between lncRNAs and genes (co-expression) were calculated with the Pearson’s correlation coefficients method (|r| > 0.95 and *p* < 0.05), as previously reported [22,30]. The cis- and trans-acting putative target genes were annotated by Gene Ontology (GO) and Kyoto encyclopedia of genes and genomes (KEGG) to explore the potential functions of lncRNAs. GO terms and KEGG pathways with a corrected *p*-value < 0.05 were defined as significantly enriched.

### 2.6. Verification of LncRNA Expression Pattern with RT-qPCR

To validate the reliability of the RNA-seq data, 10 DE lncRNAs were randomly selected to verify their expression patterns in skeletal muscle of goats at different development stages. Primers for the 10 lncRNAs and endogenous reference gene (Table 1) were designed by Primer 5, and the goat *GAPDH* was selected as the endogenous reference. Briefly, total RNA was extracted from the longissimus dorsi muscle samples. For RT-qPCR analysis, 1 μg of the total RNA was reverse transcribed using PrimeScript™ RT reagent kit (Takara, Beijing, China) according to the manufacturer’s protocol. Then, the qPCR was performed by a RocheLight Cycler^®^ 480 Ⅱ system with SYBR Green qPCR Mix kit as previous study [31]. The expression levels were analyzed using comparative threshold (2^−ΔΔ*Ct*^). All experiment were performed in triplicate. 

### 2.7. Construction of the CeRNA (lncRNA-miRNA-mRNA) Network

To further predict the functional of lncRNA in skeletal muscle development, the ceRNA (lncRNA-miRNA-mRNA) networks were constructed based on the theory that lncRNA directly associates with miRNA and subsequently affects the activity of its mRNA [32]. Firstly, the correlation between lncRNA and miRNA or miRNA and mRNA was analyzed by correlation coefficient. All pairs with COR > 0.85 and adjusted *p* < 0.05 were selected as potential lncRNA-miRNA or miRNA-mRNA pairs. Then, the ceRNA networks were constructed with the DE lncRNAs, miRNAs, and mRNAs. Finally, the networks were visualized by Cytoscape software. 

### 2.8. Statistical Analysis

RT-qPCR data and graphs were generated by GraphPad Prism 6.0 (San Diego, CA, USA). The results were presented as means ± SEMs. The unpaired two-tailed *t*-test was performed to determine the statistical difference. All experiments were performed in three replicates. A minimal standard of statistical significance was set at *p* < 0.05 or *p* < 0.01.

## 3. Results

### 3.1. RNA-Seq Data Filtering, Mapping, and Transcript Assembly

To identify the function of lncRNAs in skeletal muscle growth, two cDNA libraries were constructed using longissimus dorsi samples of goats at two development stages: 1-month-old and 9-month-old. A total of 89.57 Gb of raw data were generated. After filtering out low-quality and adaptor sequences, an average of ~14.58 Gb high-quality clean reads remained. The average GC percentage was 53.41%, with the quality scores of Q20 and Q30 above 96% and 91%, respectively (Table 2). Approximately 72.47–85.25% of the high-quality reads were mapped to the goat reference genome (Table S1). The mapped sequences in the library were assembled and a total of 65,247 transcripts were obtained.

### 3.2. Identification and Confirmation of LncRNAs in Goat Longissimus Dorsi Tissue

**Figure 2:**
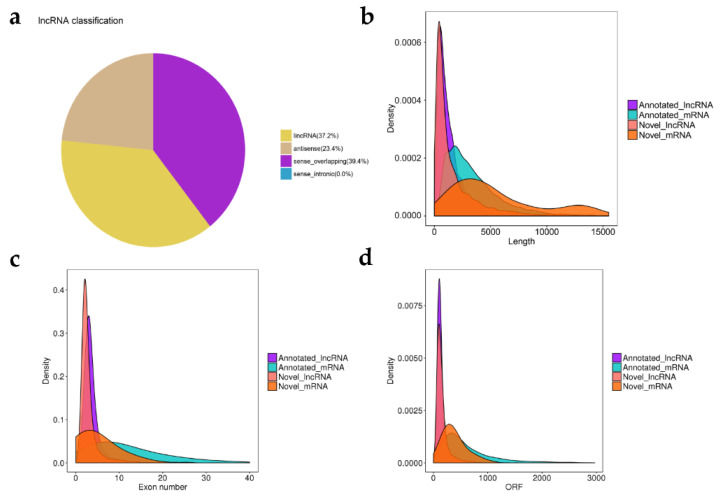
Characterization of lncRNAs. (**a**) Classification and characteristic analysis of lnRNAs. Distribution of lncRNAs transcript length (**b**), exons number (**c**), and open reading frames (ORFs) length (**d**). Annotated lncRNA, purple; Annotated mRNA, blue; Novel_lncRNA, pink; Novel_mRNA orange.

We developed a highly stringent filtering pipeline to discard transcripts that did not display characteristics of lncRNAs. The assembled transcripts from the two libraries were filtered to obtain candidate lncRNAs. A total of 3441 lncRNAs were screened (Table S2), including 1281 (37.2%) long intergenic ncRNAs (lincRNAs), 805 (23.4%) antisense lncRNAs, and 1355 (39.4%) sense_overlapping lncRNAs (Figure 2a). There was no sense_intronic lncRNAs in this study. These 3441 putative lncRNA were encoded by 2675 genes. There were 1.3 transcripts on average per locus (Table S2). The lncRNA transcripts were widespread in chromosomes, including 29 autosomes and the X chromosome (Figure S1), which reflected the function diversity of lncRNAs. 

The Illumina RNA-seq also identified 66 novel mRNAs (Table S3). As shown in Figure 2b,c, the transcript length and exon number of lncRNAs were both lower than that of the mRNA. The average length of lncRNA was 2001 bp with an average of 3.2 exons. The principal lncRNA transcripts with 2 exons accounted for 63.6% of the 3441 lncRNAs (Table S2). Importantly, the open reading frame (ORF) length of lncRNA was shorter compared with mRNA (Figure 2d). The result showed that the coding potential of lncRNAs was lower.

### 3.3. Differential Expression Analysis

**Figure 3:**
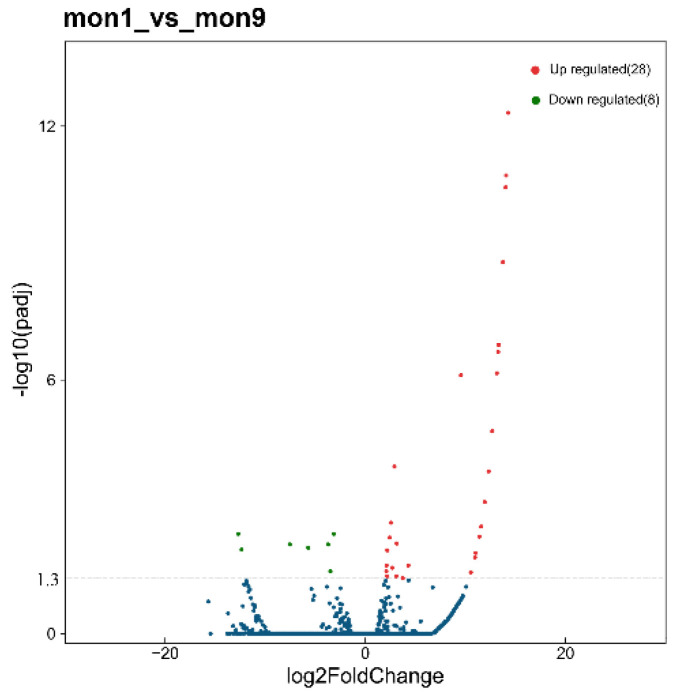
Analysis and validation of differentially expressed lncRNAs. The volcano plot analysis of all lncRNAs in 1-month-old vs. 9-month-old comparison. The red points showed the upregulated lncRNAs, the green points showed the downregulated lncRNAs, and the blue points showed the equally expressed lncRNAs.

To dissect the crucial lncRNAs involved in skeletal muscle growth in goats, we explored the DE lncRNAs (*p* < 0.05, |log2FoldChange| > 2) for 1-month-old vs. 9-month-old stage. In this study, 36 lncRNAs were differentially expressed between 1-month-old and 9-month-old stage, among which 28 were up-regulated and 8 were down-regulated at 9-month-old stage compared with 1-month-old stage (*p* < 0.05) (Figure 3, Table S4).

### 3.4. Functional Enrichment Analysis

**Figure 4:**
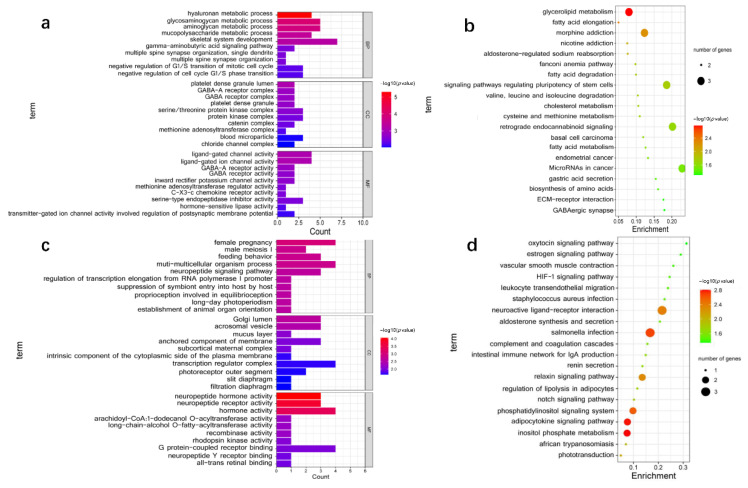
GO and KEGG [33,34,35] pathway analyses. Top 30 GO terms of the cis-acting (**a**) and trans-acting putative target genes were categorized into biological process (BP), cellular component (CC), and molecular function (MF) (**c**). Top 20 KEGG pathways of the cis-acting (**b**) and trans-acting putative target genes were shown (**d**). GO, Gene Ontology; KEGG, Kyoto Encyclopedia of Genes and Genomes.

**Figure 5:**
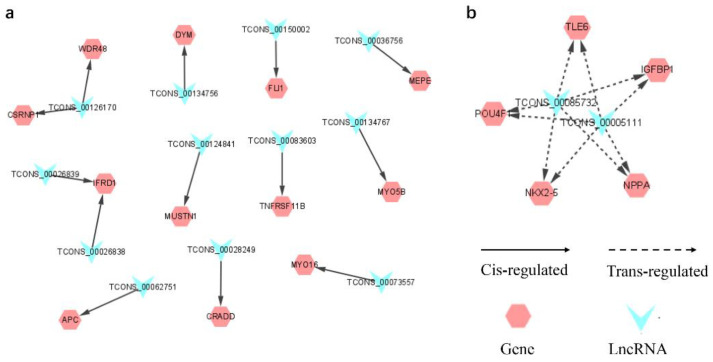
LncRNA-target gene network. The cis-target (**a**) and trans-target (**b**) networks related to muscle development-related GO terms. The V and hexagon nodes represent the DE lncRNAs and putative targeted genes, respectively. Letter abbreviations represent an abbreviated version of the gene name. Solid arrows indicate interactions between the DE lncRNAs and putative cis-target genes, while dashed arrows indicate putative trans-targets.

To evaluate the potential function of DE lncRNAs, we predicted the cis- and trans-potential targets of DE lncRNAs. In the co-location analysis, 30 lncRNAs (1 annotated lncRNAs and 29 novel lncRNAs) were transcribed close to 71 protein-coding genes (Table S5). GO enrichment analysis showed that 210 GO terms were significantly enriched (*p* < 0.05), including biological process (BP), cellular component (CC), and molecular function (MF) (Table S6). Only the top 30 GO terms were shown in Figure 4a. According to GO enrichment analysis, 12 unique genes were enriched in muscle development-related terms, such as myoblast fate determination, skeletal system development, negative regulation of G1/S transition of mitotic cell cycle, and negative regulation of cell cycle G1/S phase transition. These genes, including *IFRD1*, *CSRNP1*, *DYM*, *FLI1*, *MEPE*, *MUSTN1*, *TNFRSF11B*, *WDR48, APC*, *CRADD*, *MYO5B*, and *MYO16*, may regulate skeletal muscle development (Table S6). The co-location interactions between lncRNAs and potential target genes related to muscle development were visualized by cis-regulatory network (Figure 5a). Moreover, 57 Kyoto Encyclopedia of Genes and Genomes (KEGG) pathways were enriched through the pathway analysis (Table S7). The top 20 significantly enriched KEGG analyses were shown in Figure 4b. The potential target genes of DE lncRNAs associated with muscle development were involved in Glycerolipid metabolism, signaling pathways regulating pluripotency of stem cells, fatty acid metabolism, and biosynthesis of amino acids (Figure 4b).

Furthermore, we further predicted the potential targets of lncRNA in trans regulation based on Pearson’s correlation coefficients (|r| > 0.95). A total of 8438 interaction relationships were detected between 36 lncRNAs and 2684 mRNAs in goat reference genome (Table S8). The top 200 interaction relationships, which included 3 lncRNAs (novel lncRNAs) and 103 putative target genes, were selected for the subsequent functional cluster analysis. The putative trans-targets of DE lncRNAs were significantly enriched in 230 GO terms (*p* < 0.05) (Table S9), of which the top 30 are shown in Figure 4c. GO analysis indicated that *NKX2-5, POU4F1, NPPA, IGFBP1*, and *TLE6* were enriched in muscle development-related terms of canonical Wnt signaling pathway, muscle tissue morphogenesis, muscle organ morphogenesis, myotube differentiation, and so on. The co-expression interactions between lncRNAs and the putative target genes related to muscle development were visualized by trans-regulatory network (Figure 5b). The analysis suggested lncRNA TCONS_00085732 and TCONS_00005111 may affect muscle development through transcriptional regulation of *POU4F1* and *NKX2-5* or others. Finally, KEGG analysis indicated the putative trans-targets of DE lncRNAs were involved in inositol phosphate metabolism, phosphatidylinositol signaling system, Notch signaling pathway, and HIF-1 signaling pathway (Table S10, Figure 4d). Overall, lncRNAs and their putative target genes showed great potential in the regulation of skeletal muscle growth and development.

### 3.5. Verification of DE LncRNAs Expression Profile with RT-qPCR

**Figure 6:**
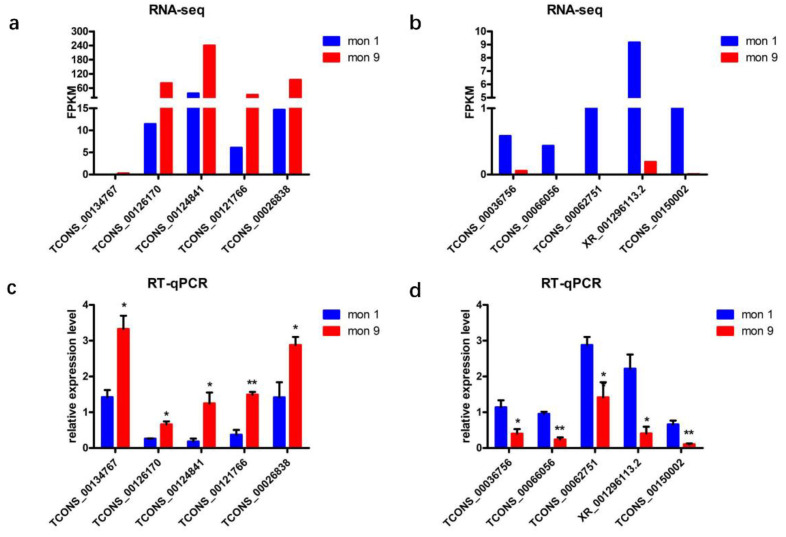
RT-qPCR validation of DE lncRNAs. The expression levels of lncRNAs downregulated in 1-month-old stage by RNA-seq (**a**) and RT-qPCR (**c**). The expression levels of lncRNAs upregulated in 1-month-old stage by RNA seq (**b**) and RT-qPCR (**d**). Data represent mean ± SE. * *p* < 0.05 and ** *p* < 0.01.

Ten DE lncRNAs were randomly selected to explore their expression profile in skeletal muscle of goats at two development stages (1-month-old, 9-month-old) using RT-qPCR. Expression analysis revealed these 10 lncRNAs exhibited differential expression profile during skeletal growth in goats (Figure 6). The expression of five lncRNAs, including TCONS_00134767, TCONS_00126170, TCONS_00124841, TCONS_00121766, and TCONS_00026838, increased during the process of skeletal muscle growth. In contrast, the expression of five other lncRNAs, including TCONS_00036756, TCONS_00066056, TCONS_00062751, XR_001296113.2, and TCONS_00150002, decreased. Importantly, the expression trends of the 10 significant differentially expressed lncRNAs were basically consistent with the RNA-seq results. The above analysis revealed that the pipeline we developed was reasonable to identify putative lncRNAs, and that the RNA-seq results were reliable.

### 3.6. Construction and Bioinformatics Analysis of the ceRNA Network

**Figure 7:**
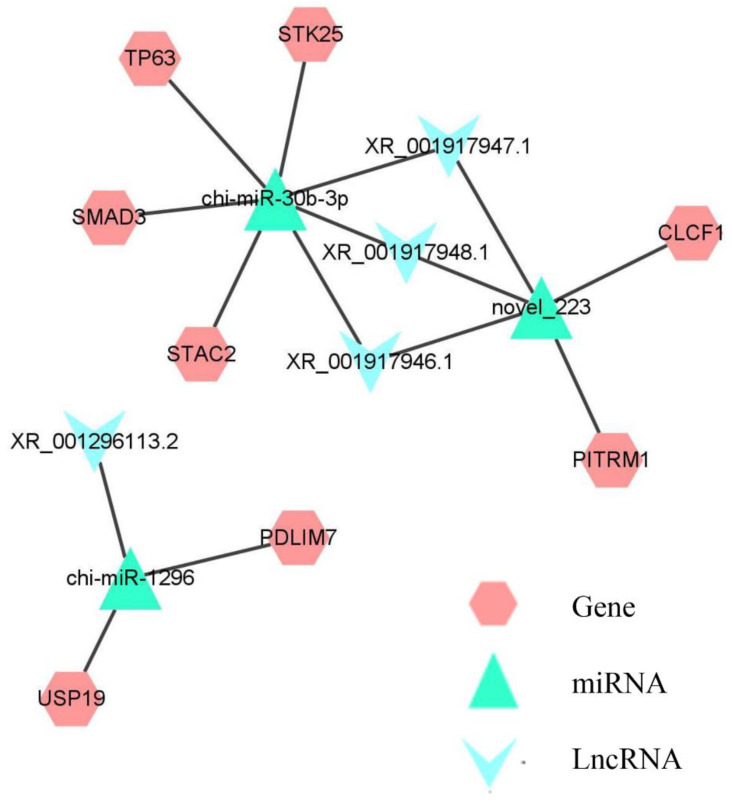
The DE lncRNA-miRNA-mRNA interaction networks. The V, triangle, and hexagon nodes represent the DE lncRNAs, DE miRNA, and DE lncRNAs targeted genes, respectively. Letter abbreviations represent an abbreviated version of the gene name.

To elucidate the potential interaction of above lncRNAs, the lncRNA-miRNA-mRNA ceRNA networks were constructed by Cytoscape software (Figure S2). Considering the functional diversity of lncRNAs, we selected lncRNAs with muscle functions, which were based on the reported roles of mRNA in previous studies to construct the following ceRNA networks diagram (Figure 7). The ceRNA networks comprised 3 miRNAs, 8 mRNAs, and 4 lncRNAs transcripts, which interacted with at least one miRNA. 

It also showed that miRNA forms the center of the network with lncRNA as the bait and mRNA as the target, suggesting that lncRNA acts as a sponge of miRNA to regulate gene expression. For instance, lncRNA XR_001296113.2 could regulate *PDLIM7* by competing with chi-miR-1296. Meanwhile, lncRNA XR_001917947.1, XR_001917946.1, and XR_001917948.1 functioned as ceRNAs by regulating chi-miR-30b-3p, which affects *smad3* expression. 

## 4. Discussion

The growth and development of skeletal muscle is one of the most important factors affecting the meat production of livestock [36]. It is well known that skeletal muscle development involves a series of exquisitely regulated and orchestrated changes in the expression of many genes [37]. Importantly, the muscle development in goats involves two main stages—the embryonic and the postnatal stages. During the embryonic stage, muscle development is completed, and the number of muscle fibers generally does not change after birth. At postnatal stage, the muscle growth is mainly triggered by muscle fiber hypertrophy (the diameter and length of the myoblast) and an increase in intermuscular fat [38]. In general, kid goat (from newborn to 90-day-old) was related to muscle fibers’ hypertrophy and regulation of myoblast proliferation [39]. In addition, goats grow very quickly from 0 to 7 months, but slowly after 18 months [40]. In this study, two growth stages (1-month-old and 9-month-old) were selected for researching the molecular mechanism of muscle growth and development. The lncRNAs expression profile of goat longissimus dorsi muscle at the two stages were detected by RNA-seq and bioinformatics analysis. We investigated the transcript structure and expression patterns of lncRNAs in goat skeletal muscle tissue, and then explored the potential functions of cis- and trans-potential target genes of lncRNAs.

Few studies have reported on the crucial function of lncRNAs expression profile in Wu’an black goat, especially in skeletal muscle growth and development. In this study, the cDNA libraries were generated with Illumina NovaSeq. An average of ~14.58Gb high-quality clean reads were obtained after the filtering process. Subsequently, the high-quality clean reads were mapped to the goat reference genome and assembled with StringTie, and a total of 65,247 library transcripts were obtained. LncRNAs can be single- or multi-exon, making it difficult to distinguish putative lncRNAs from the plentiful sing-exon, lowly expressed and unreliable sequenced fragments [41,42]. To minimize the selection of false positive lncRNAs, we set up a relatively stringent filtering pipeline to obtain true lncRNAs with high confidence. Only multi-exon lncRNAs were selected for further exploration, as was done in other studies [23,43]. As a result, 3,441 putative lncRNAs with high confidence were identified. In addition, most lncRNAs were longer than 2000 bp in length and contained 2 exons, which was in agreement with previous studies in goats and sheep [21,23]. Moreover, our current data demonstrated that lncRNAs had fewer exons and shorter transcript length and ORFs length than mRNA, which was consistent with other studies [22,23]. These similarities suggested that the putative lncRNAs verified in our study were reliable. This was essential to expand our understanding of lncRNAs via association with multiple structural features.

In recent years, numerous studies have reported on the biological functions of lncRNAs. For example, lnc-SEMT regulates *IGF2* expression via competing with miR-125b to facilitate skeletal muscle growth and development in sheep [17]. Besides, lncR125b promotes the differentiation of goat skeletal muscle satellite cells by competing with miR-125b [16]. In addition, a series of lncRNAs, such as lncIRS1, lnc-RAM, and lncMD1, have been identified to have an essential effect on skeletal myogenesis [44,45,46]. LncRNAs are non-coding transcripts that act as regulators of gene expression and are involved in skeletal muscle development [47]. In the present study, we detected 36 DE lncRNAs in skeletal muscle of goats at two growth stages. These lncRNAs showed significantly different changes between 1-month-old and 9-month-old goats. They may have certain biological functions during skeletal muscle growth. Therefore, the DE lncRNAs verified in this study could be regarded as vital putative regulators of muscle biology. Furthermore, the expression trends of 10 randomly DE lncRNA (either up or down regulated) identified by RT-qPCR were consistent with the RNA-seq results. Together, these data provided a valuable resource for exploring the role of lncRNAs in postnatal muscle growth and provided new insights into the dynamic gene regulation of muscle biology in Wu’an black goat.

Unlike protein-coding genes, the biofunctions of lncRNAs cannot be directly speculated from their sequence or structure. Therefore, we attempted to uncover the function of lncRNAs based on their potential cis- and tans-acting target genes in our study [23,39,48]. Additionally, GO and KEGG analyses were carried out to further explore the function of lncRNAs. It has been known that some lncRNAs are thought to work in cis on neighboring genes, and other lncRNAs work in trans to regulate distantly located genes. The cis-regulating target genes play an important role in estimating the biological functions of lncRNAs. We searched for the potential cis-target genes located within 100 kb upstream and downstream of the identified lncRNAs, which was comparable with previous studies [19,30]. Of these potential cis-target genes, some were all enriched in the GO term of skeletal muscle system development, including *APC*, *IFRD1*, *CSRNP1*, *TNFRSF11B*, *WDR48*, and so on. The result implied that the corresponding lncRNAs had a vital role in regulating skeletal muscle development. Emerging studies have documented that lncRNAs were associated with cis-regulation in skeletal muscle biology. For instance, the lncRNA Dum can facilitate myoblast differentiation and damage-induced muscle regeneration via silencing its cis-acting target gene, *Dppa2* [40]. Co-location correlation analysis combining GO analysis indicated that TCONS_00062751 could affect muscle development by targeting *APC.* The lncRNA TCONS_00062751 was selected because of its cis-target *APC* enriching in the GO terms of negative regulation of G1/S transition of mitotic cell cycle and negative regulation of cell cycle G1/S phase transition. Moreover, it has been reported that *APC* is required for muscle stem cell proliferation and skeletal muscle tissue repair [49]. Furthermore, the potential cis-target genes of TCONS_00026388 and TCONS_00026389 mainly enriched myoblast fate determination, in which *IFRD1* was significantly enriched. *IFRD1* played a vital role in myoblast differentiation by regulating the expression of *MyoD* and *NF-kappaB* [50]. These results suggested that lncRNAs acted in cis on neighboring protein-coding genes to regulate muscle development in goats. 

Still, some lncRNAs have been found to function in trans-acting to target gene loci far from the transcriptional location of lncRNAs [51]. For instance, LncRNA MUNC, encode 5 kb upstream of the *MyoD* transcription start site, facilitates the biofunction of *MyoD* in muscle biology [52]. In this study, the co-expression analysis between DE lncRNA and mRNA on different chromosomes were conducted based on the Pearson correlation coefficient. We found that several mRNAs were regulated by the trans-action of lncRNAs clustered in GO terms and KEGG pathways associated with muscle development. For example, *NKX2-5* was enriched in terms of negative regulation of canonical Wnt signaling pathway, muscle tissue morphogenesis, and muscle organ morphogenesis and myotube differentiation. *NKX2-5* has been reported to regulate the differentiation of skeletal myoblasts in vitro [39]. Based on the trans-regulatory interactions, TCONS_00085732 and TCONS_00005111 may affect muscle development by targeting *NKX2-5.* It was worth noting that *POU4F1* was also the potential trans-action target of TCONS_00085732 and TCONS_00005111. However, we found no reports on the regulation of muscle development by *POU4F1*. The lncRNA TCONS_00085732 and TCONS_00005111 could regulate muscle development on account of its putative trans-target *POU4F1*, mainly enriching in the GO terms of muscle tissue morphogenesis and muscle organ morphogenesis. 

Beyond that, another aspect worth considering is the low number of genes in “functionally enriched” categories (some terms have only one gene). Taking the co-location analysis as an example, the potential cis-targets of only the differentially expressed lncRNAs (not all screened 3441 LncRNAs) were predicted in the present study. As shown in the manuscript, 71 putative target genes were enriched in 210 GO terms. Therefore, the GO terms with assigned 1 gene were reasonable. Similar results have been found in several other studies [30,53,54]. The above information validated that lncRNAs may be involved in goat skeletal muscle biology through cis- or trans-regulation. Overall, these genes associated with muscle development were derived from target predictions. Further validation is necessary to explore the function of lncRNAs in skeletal muscle development in goats. 

Currently, the function roles and mechanisms of ceRNA are widely reported [55,56], in which lncRNAs act as molecular sponges that regulate the expression of target mRNAs upon binding miRNAs. In this study, the lncRNA-miRNA-mRNA ceRNA networks associated with muscle development in goats were constructed by sequencing and bioinformatic. The functional lncRNAs serve as key regulators in muscle biology [44,45,46]. Therefore, it was important to investigate the function of lncRNA in skeletal muscle growth. The ceRNA networks analysis showed that lncRNA XR_001296113.2 acted as a sponge of chi-miR-1296 to regulate the expression of *PDLIM7.* Similarly, XR_001917947.1 functioned as a ceRNA to regulate *smad3* expression by sponging chi-miR-30b-3p. The potential function of lncRNAs can be inferred from co-located or co-expressed protein-coding genes. Obviously, *PDLIM7* and *smad3* attracted our attention in this study. Previous studies have reported that *PDLIM7* is a member of the PDZ-LIM proteins family, which is known to regulate skeletal muscle development [57,58]. *Smad3* plays an important role in skeletal muscle regeneration, as lack of *smad3* signaling leads to impaired skeletal muscle regeneration [59]. Taken together, the constructed ceRNA networks may function in goat muscle. Nevertheless, the biological functions of the lncRNA-miRNA-mRNA interactions described in this study need to be validated by further studies. The discussions mentioned above all suggested that lncRNAs were an important component of the regulatory network in skeletal muscle growth and development. The bioinformatics analysis showed that these lncRNAs exhibited significant changes during skeletal muscle growth in goats, implying that they may have certain functions in myofiber growth.

## 5. Conclusions

This study used RNA-seq to systematically characterize the expression profile of lncRNAs in skeletal muscle of goats at two development stages. The DE lncRNAs associated with muscle growth in goats were validated. The verified lncRNAs in our study shared many common features in their structure. The functional annotation of DE lncRNAs was performed using GO and KEGG pathway enrichment analysis. In addition, the visualization of lncRNA-associated ceRNA networks provide a more comprehensive understanding of candidate lncRNAs that may regulate skeletal muscle growth in goats. However, the lncRNA-associated ceRNA network involved in skeletal muscle development was inferred from the protein-coding genes, and its role needs to be validated by further studies.

## Figures and Tables

**Table 1:** Primer information of RT-qPCR used in the study.

Oligo Name	Primer Sequence (5′→3′)	Product Length/bp	Annealing Temperature/°C
TCONS_00036756	F: AGGCTGCAATCCACGCTAA R: TCCAACTCTGTGTGACCCCAT	170	60
TCONS_00066056	F: AGAAAATGAATCCCTGGAGTGTG R: AACGCTGACCACCATGATGAC	114	60
TCONS_00062751	F: CTGGGAGAATACAAAGGGGG R: GGATCTACGGGCCTTTTGTCT	247	60
XR_001296113.2	F: GCCGCCGTGAAGACTATTG R: CCATGAAGCCAGGGTACAAAC	180	60
TCONS_00150002	F: CCCCGAATGTAAGCAATGAG R: AGGAGACCTACCGCTACCTGAG	145	60
TCONS_00134767	F: CCCAACAAAGTGCCCAGAC R: GGAGAAGACGGCGTTATGC	142	60
TCONS_00126170	F: GCTAGTCCCAGACAGCATTCAT R: GGTGTTGTTCTCGCCTGGAA	252	60
TCONS_00124841	F: CCCTTACCACAGGCACCACT R: CAGGTGAGAAGGTGTGTTCTGG	104	60
TCONS_00121766	F: TGTCCCCAACCTCGGTATCT R: GGTCAAACCTCTGAGCCTCG	211	60
TCONS_00026838	F: CTTCTCCTTGCTTGGCACCT R: CAGGTGCCAAGCAAGGAGA	121	60
GAPDH	F: CACGGCACAGTCAAGGCAG R: AGATGATGACCCTCTTGGCG	196	60

**Table 2:** Data summary of RNA-seq in goat muscle.

Sample Name	Raw Reads	Clean Reads	Raw Bases (G)	Clean Bases (G)	Error Rate (%)	Q20 (%)	Q30 (%)	GC Content (%)
mon1_1	98721676	97036650	14.81	14.56	0.03	97.47	93	52.99
mon1_2	104194360	101986738	15.63	15.3	0.03	97.29	92.64	55.08
mon1_3	99110136	97525086	14.87	14.63	0.03	96.95	91.87	53.06
mon9_1	98408274	96645866	14.76	14.5	0.03	97.4	92.79	51.64
mon9_2	90534786	88937344	13.58	13.34	0.03	97.48	92.97	53.35
mon9_3	106156574	101082860	15.92	15.16	0.03	96.92	91.93	54.31

## Data Availability

The datasets supporting the conclusions of this article are included within the article and its supplementary files. The raw data obtained from RNA-seq can be found in the National Center for Biotechnology Information (NCBI) Sequence Read Archive repository, https://www.ncbi.nlm.nih.gov/sra/PRJNA749569 (accessed on 24 August 2022).

## Supplementary Materials

The following supporting information can be downloaded at: https://www.mdpi.com/article/10.3390/ani12192683/s1, Figure S1: Chromosome distribution of lncRNAs identified in goat skeletal muscle.; Figure S2: The lncRNA-miRNA-mRNA ceRNA networks diagram.; Table S1: The information of RNA-seq data.; Table S2: List of lncRNAs identified in goat skeletal muscle libraries.; Table S3: List of mRNAs identified in goat skeletal muscle libraries.; Table S4. List of DE lncRNAs from two muscle developmental stages.; Table S5: Protein-coding genes detected 100 kb upstream and downstream of DE lncRNAs.; Table S6: GO enrichment analysis of protein-coding genes co-located with lncRNAs.; Table S7: KEGG analysis of protein-coding genes co-located with lncRNAs.; Table S8: Co-expression analysis between protein-coding genes and lncRNAs.; Table S9: GO enrichment analysis of protein-coding genes co-expressed with lncRNAs.; Table S10: KEGG analysis of protein-coding genes co-expressed with lncRNAs.
