# Supplementary figures and images for: Identification and Characterization of lncRNAs Expression Profile Related to Goat Skeletal Muscle at Different Development Stages
Source: Animals (Basel). 2022 Oct 6;12(19):2683. doi: 10.3390/ani12192683 (PMC9558979; doi:10.3390/ani12192683)

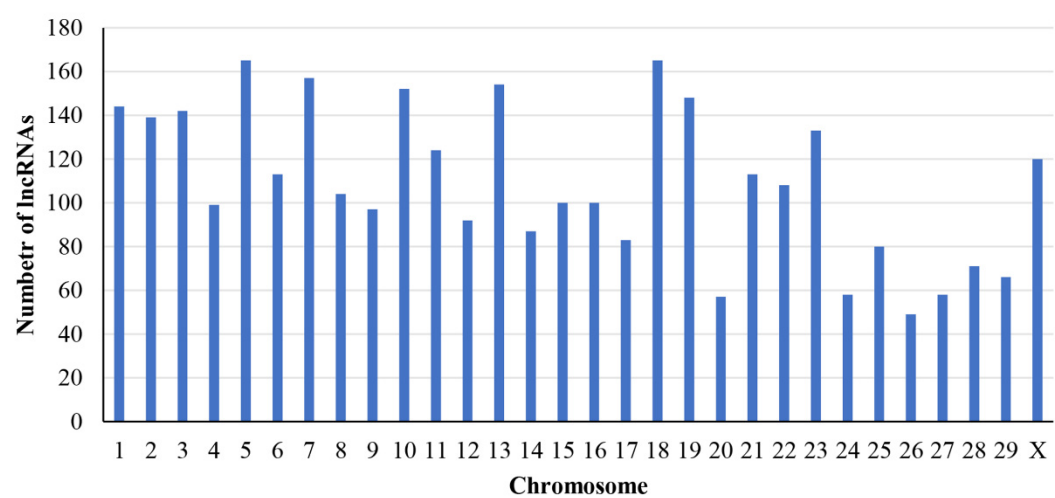

**Figure S1.** Chromosome distribution of lncRNAs identified in goat skeletal muscle.

Supplement: Supplementary file 1 [file animals-12-02683-s001.zip › Supplementary FigureS1.pdf]

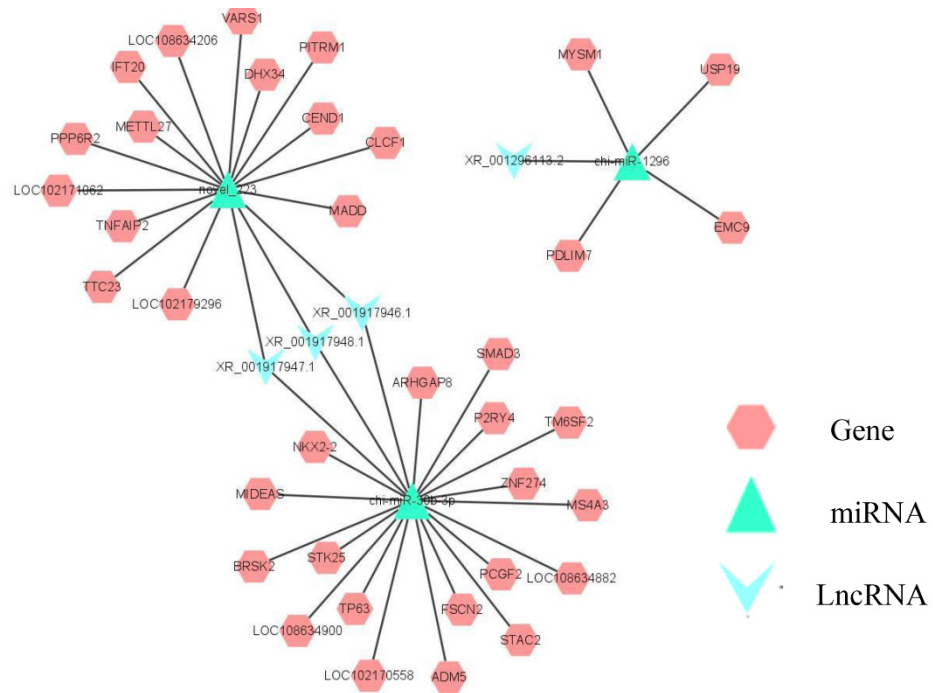

**Figure S2** The lncRNA-miRNA-mRNA ceRNA networks diagram.

Supplement: Supplementary file 1 [file animals-12-02683-s001.zip › Supplementary FigureS2.pdf]
